# “Head-to-Side-Chain” Cyclodepsipeptides of Marine Origin

**DOI:** 10.3390/md11051693

**Published:** 2013-05-21

**Authors:** Marta Pelay-Gimeno, Judit Tulla-Puche, Fernando Albericio

**Affiliations:** 1 Institute for Research in Biomedicine (IRB Barcelona), Baldiri Reixac 10, Barcelona 08028, Spain; 2 CIBER-BBN, Networking Centre on Bioengineering, Biomaterials and Nanomedicine, Baldiri Reixac 10, Barcelona 08028, Spain; 3 Department of Organic Chemistry, University of Barcelona, Martí i Franqués 1-11, Barcelona 08028, Spain; 4 School of Chemistry and Physics, University of KwaZulu-Natal, Durban 4001, South Africa

**Keywords:** natural products, peptides, polyketides, therapeutic agents

## Abstract

Since the late 1980s, a large number of depsipeptides that contain a new topography, referred to as “head-to-side-chain” cyclodepsipeptides, have been isolated and characterized. These peptides present a unique structural arrangement that comprises a macrocyclic region closed through an ester bond between the *C*-terminus and a β-hydroxyl group, and terminated with a polyketide moiety or a more simple branched aliphatic acid. This structural pattern, the presence of unique and complex residues, and relevant bioactivity are the main features shared by all the members of this new class of depsipeptides, which are reviewed herein.

## 1. Introduction

Since ancient times, humans have used natural products to treat nearly every ailment known [1]. Furthermore, for the last few decades, natural products have also served as biochemical tools to better understand the targets and pathways involved in disease. Their structural complexity, chemical diversity, and biological specificity are matchless. Thus, the chemical space that these products represent differs to that of synthetic compounds and cannot be easily replaced [2].

However, the use of natural products as the main source for drug discovery also has a number of severe intrinsic drawbacks. Firstly, labor-intensive and time-consuming procedures are needed to obtain the amounts required for pre-clinical studies. Secondly, these products have an extremely high structural complexity, thus representing a synthetic challenge and a considerable obstacle for the development of analogs. Thirdly, the use of natural sources may lead to the re-discovery of already known compounds. Finally, the time-line for the development of drugs from natural products exceeds the standards set by pharmaceutical companies [3].

In the early 1990s, the development of High-throughput screening (HTS), together with the introduction of combinatorial chemistry, pushed natural product research programs into the background [4]. The higher capacity to produce large libraries of new synthetic compounds in a short time by means of combinatorial chemistry and the dramatic increase in the speed of the biological evaluation thanks to HTS refinement were unbeatable improvements with respect to the disadvantaged screening of natural products. As a result, pharmaceutical companies significantly reduced their drug discovery programs based on natural products [5].

However, to date, only one *de novo* combinatorial NCE (New Chemical Entity) has been approved by the FDA, namely the kinase inhibitor sorafenib for renal carcinoma [6,7]. Thus, the failure of large synthetic libraries in the race to find first-in-class drugs combined with several improvements in the screening of natural products have enabled a small revival of natural products in drug discovery programs in recent years.

Simultaneously, the so-called chemistry of natural products has been transformed by new methodologies and technological advances, which have facilitated the re-establishment of natural products as the main pipeline for drug development. Thus, the combination of HPLC and SPE (solid phase extraction) techniques with NMR and MS technologies and the implementation of bioassay-guided fractionation have substantially decreased the time required for dereplication, isolation and structure elucidation. As a result, the screening process for natural products has become more efficient and straightforward.

Furthermore, several breakthroughs have been made in synthetic organic methodologies in the last few years: the development of the semi-synthetic approach, consisting of the generation of analogs by synthetically modifying natural products [8,9]; the introduction of the chemoenzymatic approach, which uses not only chemical tools, but also enzymes to transform natural products [10,11]; the application of diverted total synthesis [12]; the use of combinatorial chemistry tools to build libraries based on natural product scaffolds [2,13,14]; and combinatorial biosynthesis, which uses genetic engineering to create libraries based on natural products [15,16]. All these achievements have enabled the chemist to access increasingly more complex structures [3].

Moreover, advances in microbial genomics may bring about a revolution in the discovery of natural products [17]. This new technology has enabled the rapid identification of genes encoding novel secondary metabolites (most of which would have probably gone unnoticed by traditional screening methods) and the computational prediction of chemical structures on the basis of genomic sequences. Furthermore and more importantly, it is now possible to establish specific fermentation conditions to express genes encoding a particular natural product [18,19].

In this new drug discovery panorama, the use of marine actinomycetes, fungi and myxobacteria as sources of natural products could represent a more prolific approach for the development of drugs based on natural structures, thus limiting the chances of finding known compounds [20,21,22]. 

In this regard, the marine ecosystem occupies 70% of the earth’s surface and holds a huge biodiversity, which is subjected to higher evolutionary pressure and a smaller impact of humans than the terrestrial ecosystem. Thus, hosting unprecedented, highly diverse and extremely complex structures with a range of biological activities, the marine ecosystem is a potent reservoir of natural compounds [23]. And until only 30 years ago, it was still unexplored. Technological improvements in scuba diving and harvesting procedures, together with the development of more accurate and precise chromatographic techniques, that enable the identification and biological evaluation of new natural compounds using smaller amounts of products, have facilitated a more exhaustive exploration of the marine environment for drug candidates. Thus, since the 1970s, more than 15,000 natural products with distinct bioactivities have been isolated from marine microbes, algae and invertebrates [24].

Marine life has adapted to and survived the most extreme conditions, such as fierce interspecies competition over limited resources, sub-zero temperatures, and almost a complete lack of oxygen and light. Moreover, most marine organisms are not provided with physical defense systems to protect themselves against predators, to feed and to safeguard their own space. Hence, they have developed highly efficient and sophisticated chemical tools that serve as an “immune” system. By excreting these chemicals into the environment, they protect themselves from external aggression. The “dilution effect”, which occurs when these secondary metabolites are released into the sea water, guarantees the survival of only the most effective chemicals, turning them into the most attractive natural compounds for consideration in biomedical programs.

Natural marine compounds have interesting and diverse biological profiles, including bioactive properties, such as anti-tumor, anti-cancer, anti-microtubule, anti-proliferative, anti-hypertensive, anti-inflammatory, anti-virus, anti-fungal, cytotoxic, and antibiotic activity [25].

**Figure 1:**
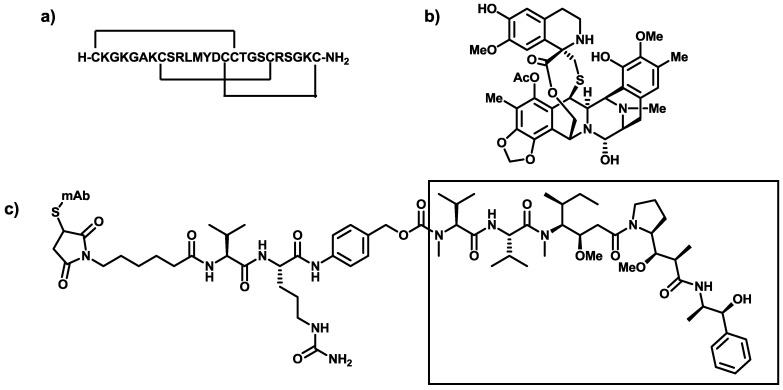
Chemical structures of (**a**) Ziconotide; (**b**) Trabectedin; and (**c**) Brentuximab vedotin (the box contains the moiety of marine origin), which illustrate the chemical diversity found in the sea.

The efforts channeled into drug development from marine sources have started to bear fruits. Thus, in December 2004, Ziconotide (known under the trade name of Prialt and developed by Elan Pharmaceuticals) [26] was the first drug derived from a natural marine product approved in the US for the treatment of pain. It is the synthetic form of ω-conotoxin MVIIA, a linear 25-amino acid peptide isolated from the venom of a tropical marine cone snail. This peptide shows a well-defined three-dimensional structure stabilized by the presence of three disulphide bridges. Moreover, in October 2007, Trabectedin (known under the trade name Yondelis and developed by PharmaMar) [27] became the first marine anti-cancer drug to receive approval in the EU. This drug is an alkaloid isolated in extremely poor yields from extracts of the Caribbean tunicate *Ecteinascidia turbinata* and accessed by means of a semi-synthetic approach from the multi-kilogram scale antibiotic cyanosafracin B. Finally, Brentuximab vedotin (known under the trade name Adcetris and developed by Seattle Genetics) [28] was approved by the FDA in August 2011 for the treatment of Hodgkin’s lymphoma and systemic anaplastic large cell lymphoma (ALCL). From a structural perspective, it consists of three components: a specific antibody for human CD30 (chimeric immunoglobulin G1 mAb cAC10) covalently linked to a synthetic analog of the natural marine product dolastatin 10 (the microtubule-disrupting agent MMAE) through a protease-cleavable linker (Figure 1).

The role and potential of natural products in drug discovery programs are reaffirmed by the following recently published figures. Data from 1981 to 2010 show that, of all the drugs approved, 60% were either natural products or based on natural structures. Moreover, when specifically referred to anti-cancer drugs, this percentage increased to 70% [29]. These data, together with the significant number of drugs derived from natural products and currently undergoing evaluation in clinical trials [30], illustrate the importance of natural sources for the development of new drugs, even when many research programs in natural product discovery run by pharmaceutical companies have been terminated.

Natural marine products are of highly diverse chemical nature: phenols, alkaloids, terpenoids, polyesters, peptides, and other secondary metabolites produced by marine organisms, such as sponges, bacteria, dinoflagellate and seaweed. Several reviews about marine peptides and depsipeptides have been published so far [31,32,33,34]. However, the present review focuses on a particular class of marine peptides, the “head-to-side-chain” cyclodepsipeptides, most of which display interesting therapeutic activity.

## 2. “Head-to-Side-Chain” Cyclodepsipeptides

A peptide is an oligomer formed by up to 60 amino acids. When at least one of the amide bonds of a peptide is replaced by an ester bond, we refer to depsipeptides. These structures have proven to be highly promising candidates for pharmaceutical agents because they show huge structural diversity and display a broad spectrum of biological properties: anti-plasmodial, anti-viral, anti-microbial, insecticidal, cytotoxic, anti-proliferative and anti-cancer activities. Furthermore, they can also show ionophoric and anthelmintic properties. Most bioactive peptides have been extracted mainly from tunicates, bacteria, sponges and mollusks [31].

The presence of several unique residues and the complex structural features shown by marine depsipeptides makes the structural elucidation of these compounds a challenge. However, their interesting therapeutic profiles have boosted research in this field, resulting in several depsipeptides now undergoing evaluation in clinical trials.

Since the late 1980s, a number of depsipeptides have been reported that belong to a new family of peptides, namely “head-to-side-chain” cyclodepsipeptides. These peptides present a unique structural arrangement: a macrocyclic region closed through an ester bond between the *C*-terminus and a β-hydroxyl group, terminated with a polyketide moiety or a more simple branched aliphatic acid. This structural pattern, the presence of unique and complex residues and interesting bioactivity are the main features shared by all the members of this new class of depsipeptides.

## 3. Families of “Head-to-Side-Chain” Cyclodepsipetides

**Figure 2:**
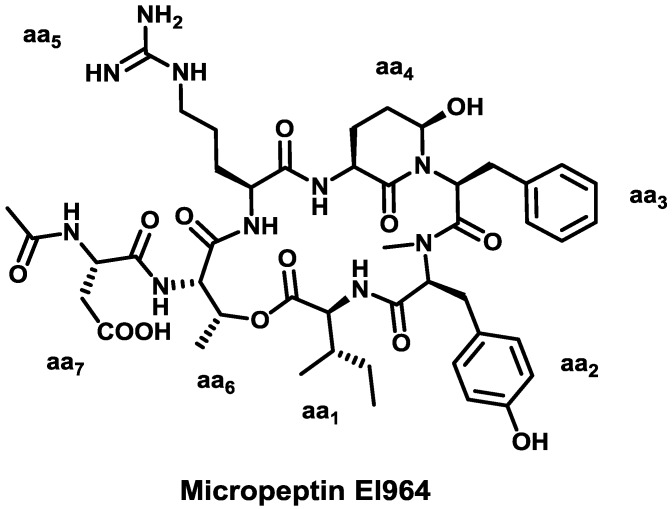
Chemical structure of micropeptin EI964.

With a wide range of structures and biological profiles, marine cyanobacteria are an extremely prolific source of natural compounds. Among all the secondary metabolites produced by these organisms, we wish to highlight the 3-amino-6-hydroxypiperidone (Ahp)-containing cyclodepsipeptides micropeptins [35,36], oscillapeptins [37,38], nostopeptins [39,40], aeruginopeptins [41,42], largamides [43], and lyngbyastatins [44,45]. These are all cyclodepsipeptides with serine protease inhibitory activity, and they share the same structural scaffold: a “head-to-side-chain” macrocyclic region, mostly comprising six residues, closed through an ester bond with the hydroxyl group of a l-Thr, and terminated with a more mutable linear fragment, often blocked by hydrophobic acids. Micropeptin EI964 [46] exemplifies this family of cyclodepsipeptides (Figure 2).

Commonly, aa_1_ is l-Ile, l-Val or l-Leu (all hydrophobic residues); aa_2_ is *N*Me-l-Tyr, which can present a number of substitution patterns (halogenated, *N*Me-l-Tyr(OMe)…) or *N*Me-l-Phe; aa_3_ accepts a higher variability and can be a hydrophobic residue such as l-Ile, l-Phe, l-Val and l-Leu or a polar uncharged amino acid such as l-Thr; aa_4_ is always Ahp or its methylated version Amp; and aa_5_ is the residue showing the highest diversity, and it can be l-Leu, Abu, l-Arg, l-Tyr, l-HomoTyr, l-Lys, l-Gln, l-HcAla, or l-Trp. The macrolactone framework represents a potent inhibitor prototype in which aa_5_ binds the S1 specificity pocket of the enzymes, thus blocking the interaction of proteases with their substrates [47,48]. Thus, the nature of this residue is crucial in terms of selectivity and explains its variability. Trypsin shows preference for basic residues (Arg and Lys) while chymotrypsin has preference for large hydrophobic residues (Tyr, Phe, Trp) [47,49,50,51,52]. Abu enhances selectivity for elastase [45]. The *O* from the hydroxyl group of Ahp/Amp participates in an intramolecular hydrogen bond with the NH of aa_1_ and the *N*Me-Tyr or *N*Me-Phe facilitates the formation of a *cis* peptide bond. These structural features make the cycle rigid [47,50,51], thus protecting this class of cyclodepsipeptides from protease activity and providing a clearly-defined 3D structure. The presence of unnatural amino acids in their structures also confers resistance to enzymatic hydrolysis.

The l-Thr residue at the branching position plays a crucial structural role and has been described to occupy the subsite S2 of the protease. Only a few nostopeptins present a different amino acid at this position, a 3-hydroxy-4-methylproline residue, whose stereochemistry has not been assigned to date [53].

The linear arm is postulated to interact with more distal regions of the proteases (S3 and S4 subsites) through hydrogen bonds [37]. Hence, selectivities and potencies can also be influenced by the highly diverse length and nature of the exocyclic fragment. Experimental data showed the requirement of at least a two unit-long exocyclic arm for strong activity [54].

From a synthetic point of view, only a few members of this huge Ahp-containing depsipeptides family have been synthesized. Thus, somamide A [55] was fully synthesized in solution while a full solid phase strategy was developed for symplocamide A [56,57]. The key step of symplocamide A synthesis is the spontaneous formation of the Ahp moiety from a glutamic aldehyde generated once the linear precursor is totally assembled. The ester bond is formed after incorporation of the two exocyclic moieties using DIPCDI-DMAP (10:1). The peptidic chain is elongated through the ester bond using Boc chemistry to prevent DKP formation at the ester level and the macrolactamization step is performed on solid phase.

A closely related “head-to-side-chain” arrangement was also found in another family of cyclodepsipeptides. **Kahalalides** [58,59,60,61,62] were isolated from the herbivorous marine mollusks *Elysia rufescens*, *E. ornate* or *E. grandifolia* and their algal diet *Bryopsis pennata* or *B. plumosa*. These compounds make up a large family of marine peptides with highly variable traits. Its members range from tripeptides to tridecapeptides and include homodetic linear peptides, “head-to-tail” cyclodepsipeptides, and “head-to-side-chain” cyclodepsipeptides, all of these acylated at their *N*-terminus.

**Figure 3:**
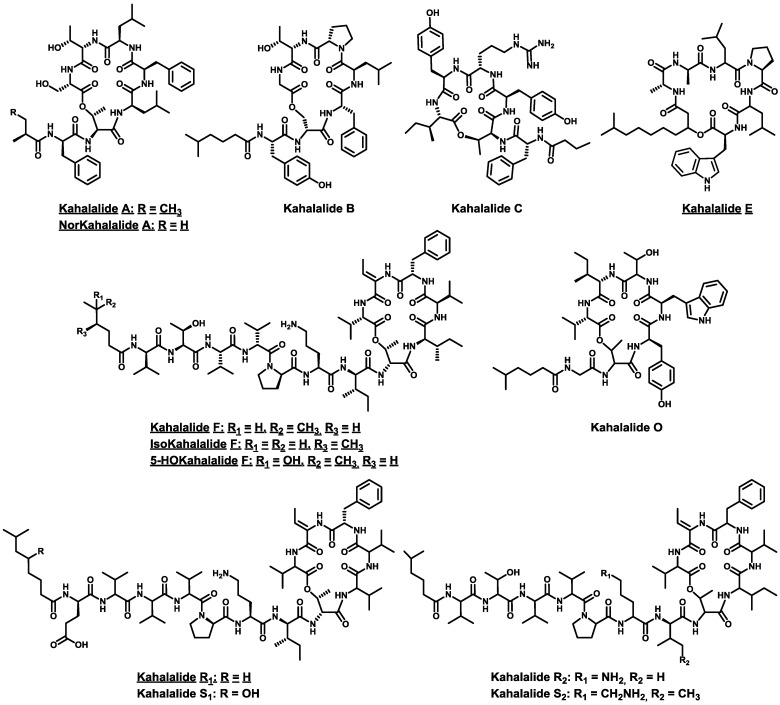
Structures of “head-to-side-chain” kahalalides plus kahalalide E. All the kahalalides displaying biological activity of interest are underlined.

Among the 24 natural members of this family described to date, only 7 show therapeutic profiles of interest [58,59,63,64,65,66,67]. These exhibit highly diverse biological properties, including cytotoxic and anti-tumor, anti-microbial, anti-leishmanial and immunosuppressive activities. Of note, all the active members of this small group are cyclodepsipeptides and 6 out of the 7 exhibit the uncommon “head-to-side-chain” structural arrangement closed through an ester bond (Figure 3).

From a structural perspective, our interest focuses on “head-to-side-chain” kahalalides, meaning kahalalide A, B, C, F, O, R_1_, R_2_, S_1_, S_2_, norkahalalide A, isokahalalide F and 5-OHkahalalide F. The macrocyclic region of these compounds comprises 6 amino acids, with one exception, kahalalide C, which bears a 5-residue ring. Most of the amino acids found on kahalalides have hydrophobic or polar uncharged side chains with an abundance of l- and d-configurations. This feature differs greatly from the Ahp-containing depsipeptide family. Other differences between the two large families are as follows: the presence of other residues such as d-Ser or d-*allo*-Thr, and not only l-Thr, at the branching position of the kahalalides; the commercial availability of all their residues, with the exception of Abu; the absence of *N*Me amino acids; the occurrence of a much longer exocyclic arm (up to seven residues); and the presence of a much simpler *N*-terminal acid, which is generally completely aliphatic and only in some cases a hydroxy acid.

However, as reported for the Ahp-containing depsipeptides, the “head-to-side-chain” structural arrangement also seems to be essential to preserve the biological activity of the kahalalides with therapeutic interest.

Kahalalide F shows the most complete biological activity profile of all the natural kahalalides described so far [58,59,65,66,67]. It shows selectivity against solid tumor cell lines with IC_50_ in the low μM range (in A-549, HT-29, LOVO, P-388, KB and CV-1 cells); antiviral activity against HSV II using mink lung cells; anti-fungal activity with IC_50_ values below 3.3 μM against *Candida albicans*, *C. neoformans* and *Aspergillus fumigatus*; immunosuppressive activity; and anti-leishmanial activity with good to moderate IC_50_ values against *Leishmania donovani* and *L. pifanoi* (promastigotes and amastigotes). Thus, exhaustive structural, synthetic, biological, clinical and mechanistic studies have been conducted over the last 20 years.

A complete analogs program has provided valuable data on structure-activity relationships (SARs) [66,67]. Kahalalide F holds three domains: domain A contains the macrocyclic region; domain B comprises the linear exocyclic arm; and domain C is formed by the *N*-terminal acid. Several changes were made in all the domains to construct 143 analogs. It was concluded that the ring is crucial to preserve the biological properties, as the linear precursor of KF, kahalalide G, did not display any activity; the presence of the Z-Dhb residue and several d-aa increase resistance to enzymatic hydrolysis and provides rigidity, which is essential to conserve the activity; the position occupied by l-Phe must host a highly hydrophobic residue; the stereochemical information of all the amino acids is critical, meaning that the peptide is highly three-dimensionally structured; and, finally, an increase in the sterical hindrance and/or the hydrophobic nature of the side chains increases activity.

To further support the experimental data collected for kahalalide F, a more reduced analogs program conducted for kahalalide A [68] provided similar conclusions regarding the “head-to-side-chain” arrangement. The synthesis and biological evaluation of 5 analogs, including its linear version, was performed to determine the importance of the macrolactone framework and the presence of free Ser and Thr residues to conserve its activity against *Mycobacterium tuberculosis*.

From a synthetic point of view, only total synthesis of kahalalide A [68], B [69], and F [70,71,72] have been published. Kahalalide A was accessed by means of total synthesis on solid phase. The Kenner sulfonamide safety-catch linker was used to cyclize the linear precursor. The ester bond over the l-Thr residue was built once the two moieties of the exocyclic arm had been assembled. The Fmoc-l-Ser(*^t^*Bu)-OH was incorporated following a double treatment with DIPCDI and DMAP (4:4:0.4) in THF (2 h + o/n).

Kahalalide B was synthesized following two synthetic strategies: complete elongation of the linear precursor on solid phase and cyclization through: (i) the peptidic bond Thr-Gly; or (ii) the ester bond d-Ser-Gly. The first approach has some advantages: a faster cyclization reaction; a cleaner and better yielding cleavage step; and the avoidance of a last deprotection step in solution. Cyclization through the ester bond has not been attempted for any other related cyclodepsipeptide. Only the presence of the least bulky β-hydroxy-α-amino acid at the branching position (d-Ser) allows such a synthetic approach.

Much effort has been channeled into the synthesis of the more complex kahalalide F. Thus, not only a linear solid phase synthesis but also convergent strategies have been successfully developed. The synthesis of linear kahalalide F was started by anchoring Fmoc-d-Val-OH onto 2-CTC resin. The ester bond was formed before complete assembly of the exocyclic arm by means of DIPCDI-DMAP. The *Z*-Dhb residue was generated on solid phase or in solution and incorporated as a dipeptide. The final cyclization step was carried out between the residues d-Val-Phe. The convergent strategies comprise the solid phase syntheses of branched peptides using tri- and tetra-orthogonal protection schemes; their cyclization and deprotection of the *N*-terminal in solution; the synthesis on solid phase of the linear components; and the final condensation of the two fragments in solution using PyAOP-DIEA. The best approach condenses the fragments through the peptidic bond d-Pro-Orn to prevent epimerization. No DKP formation at the ester level was detected.

Didemnins [73,74,75] and tamandarins [75,76] are complex cyclodepsipeptides isolated from Caribbean and Brazilian tunicates respectively. These compounds show relevant anti-tumor, anti-viral and immunosuppressive activities at low nano- and femto-molar range. Several clinical trials of some of the most valuable didemnins are still underway [77,78,79]. They have a “head-to-side-chain”-like structural arrangement closely related to those described previously, but comprising two ester bonds.

**Figure 4:**
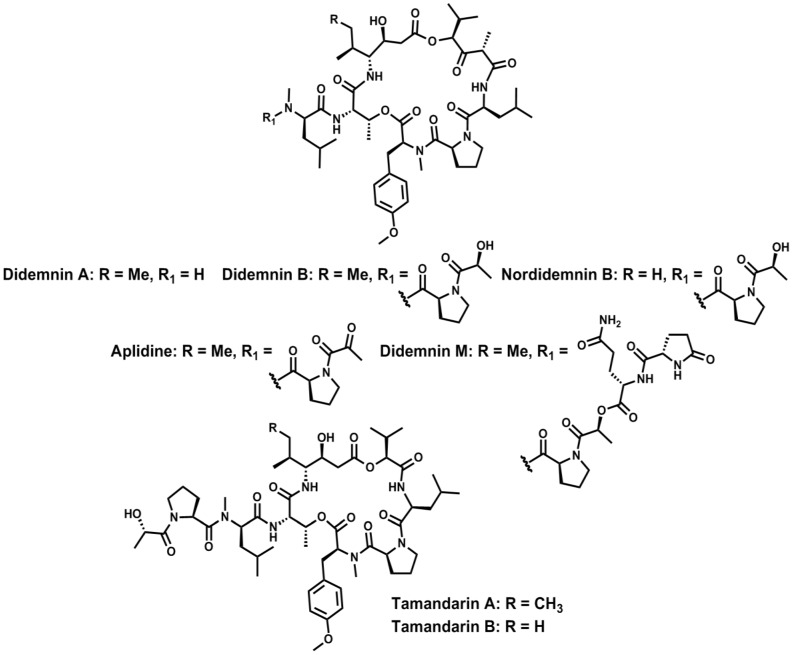
Structures of natural didemnins and tamandarins.

From a structural perspective, tamandarins are simplified versions of didemnins (Figure 4). They contain a γ-amino acid and a α-hydroxy acid (*S*-hydroxyisovaleric acid, Hiv). The Hiv moiety forms the second ester bond in the macrocyclic region and, in didemnins, it is replaced with the more complex α-(α-hydroxyisovaleryl)propionyl (HIP) unit. Both types of cyclodepsipeptides contain l- and d- and *N*Me amino acids and, importantly, as occurs in the Ahp-containing cyclodepsipeptide family, l-Thr always occupies the branching position.

Several total syntheses for didemnins A [80,81,82], B [80,81,82] and C [80,82] and dehydrodidemnin B [83] and nordidemnin B [84] have been developed. These were all synthetic approaches in solution, differing mainly in the site of cyclization and the coupling reagents used [85]. Furthermore, a number of total syntheses in solution of tamandarin A [86,87], tamandarin B [87,88] and analogs [89,90,91] have also been described. Significantly, once again, structure-activity relationships of didemnins indicate that the macrolactone framework is a crucial structural arrangement regarding the bioactivity of the natural peptides, especially cytotoxicity and anti-viral properties [92].

**Figure 5:**
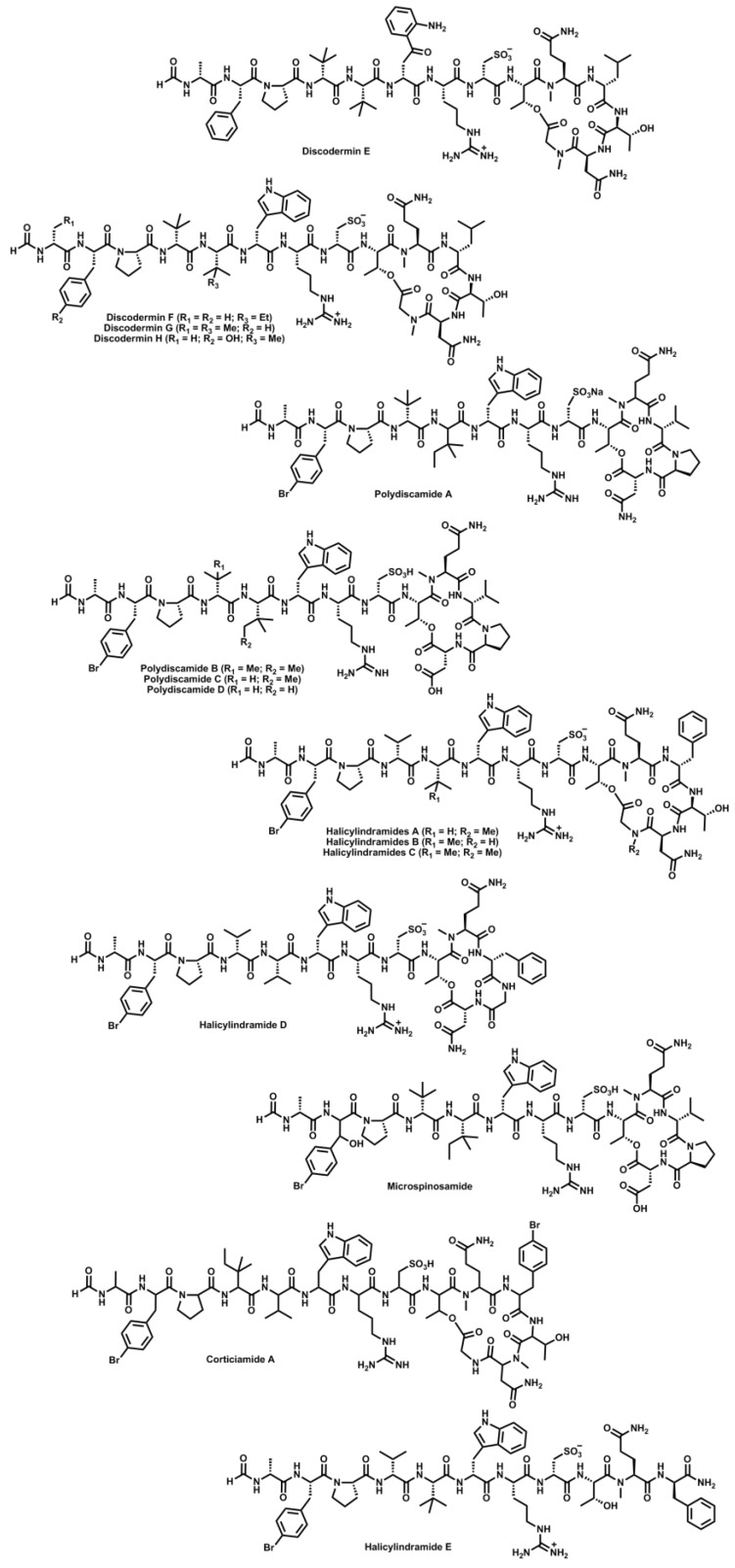
Structures of discodermins, polydiscamides, halicylindramides, microspinosamide and corticiamide.

Discodermin A [93], a tetradecapeptide displaying antimicrobial activity and inhibiting the development of starfish embryos, was firstly isolated from the marine sponge *Discodermia kiiensis* in 1984. A large number of “head-to-side-chain” cyclodepsipeptides with extremely high structural similarity have been isolated since then from different marine sponges. Thus, discodermins A–H [94,95,96,97] from *Discodermia kiiensis* marine sponge; polydiscamides A [98] also from a *Discodermia* sponge found in the Caribbean and polydiscamides B–D [99] from a sponge *Ircinia* sp.; halicylindramides A–E [100,101] from *Halichondria cylindrata*; microspinosamide [102] from *Sidonops microspinosa*; and corticiamide A [103] from the sponge *Corticium* sp. were isolated and then elucidated and described in the literature. They are either tetradecapeptides comprising a 19-membered macrolactone, or tridecapeptides containing a 16-membered macrolactone, with the *N*-terminus formylated in all cases (Figure 5). The branching position of all these peptides is occupied by an l-Thr moiety, which is always acylated by the uncommon d-cysteic acid. Furthermore, they comprise l- and d-residues and one *N*Me amino acid, and share a number of rare moieties such as l-*p*-Br-Phe, l-*t*-Leu, l-β-MeIle and Sar. Their arrangement combining a small cyclic region with a long exocyclic arm, evokes kahalalide F. From a biological perspective, they display mostly cytotoxic and antimicrobial activities. However, polydiscamides B–D have been described to act as SNSR (sensory neuron-specific G protein couple receptor) agonists and microspinosamide has been reported to inhibit the cytopathogenic effect exerted by the HIV-1 virus. Corticiamide A has not been biologically tested and the stereochemical information of its constitutive moieties has not been determined yet. Finally, halicylindramide E, which is a truncated and linear version of halicylindramide B amidated at its *C*-terminus, loses the cytotoxicity and shows only a moderate antifungal activity, significantly lower than the one exhibited by the cyclic halicylindramides. This experimental data reinforces the hypothesis of the “head-to-side-chain” arrangement being crucial for the bioactivity displayed by these peptides. The synthesis of halicylindramide A has been published by Seo and Lim [104]. They describe a fully solid-phase approach starting by the side-chain incorporation of a l-Asp moiety onto the Rink amide resin. The linear precursor is assembled until the cysteine amino acid before performing the macrolactamization step through the l-Asn-Sar linkage. Then, the exocyclic arm is elongated, the cysteine oxidized to cysteic acid and the *N*-terminus blocked with a formyl moiety.

Finally, there is a huge and varied family of cyclodepsipeptides, all produced by marine sponges, that also have the distinctive “head-to-side-chain” structural arrangement *via* an ester bond. All of them contain a 22- or a 25-membered macrolactone that bears a β-hydroxy-α-amino acid with the d-*allo* configuration at the branching position. Furthermore, they share a number of uncommon residues described for the first time after the isolation of these natural peptides. Of note, the same rare residue (diMe-Gln) acylates the branching position of all the isolated members. The exocyclic arm is either composed of three amino acids, one being a γ-amino acid, or four α-amino acids. Its *N*-terminus is always acylated with a β-hydroxy acid. Both l- and d- residues, as well as *N*Me amino acids, are found in the structures of these peptides. Finally, all the cyclodepsipeptides show therapeutic profiles of interest, displaying mostly cytoprotective activity against HIV-1 infection.

**Figure 6:**
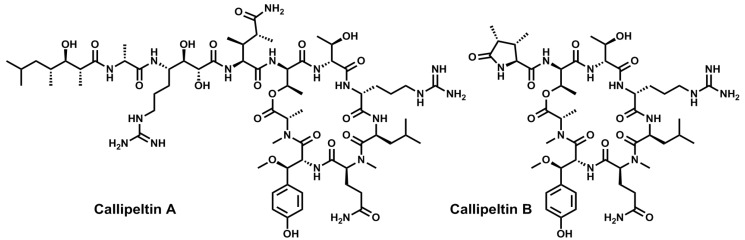
Chemical structures of callipeltins A and B. The stereochemistry of all chiral centers has been checked and corrected.

In 1996, Zampella and co-workers described the first member of the callipeltins family. Callipeltin A [105] is a “head-to-side-chain” cyclic depsidecapeptide isolated in the waters off New Caledonian from a collection of marine sponges of the genus *Callipelta*, in the order *Lithistida*. Its isolation allowed the description of three new and complex amino acids, namely β-methoxytyrosine, (3*S*,4*R*)-3,4-dimethyl-l-glutamine, and (2*R*,3*R*,4*S*)-4-amino-7-guanidino-2,3-dihydroxyheptanoic acid. From the same marine sponge, a truncated version of callipeltin A named callipeltin B, and the linear peptides callipeltins C–M were also isolated [106,107,108,109]. Callipeltins A and B (Figure 6) contain a d-*allo*-Thr at the branching position of a 22-membered macrolactone (6 residues) and display relevant cytotoxicity against several cancer cell lines. Additionally, callipeltin A was found to be active against HIV virus.

The total synthesis of callipeltin B on solid phase was published by Lipton and co-workers [110]. They chose to start the linear peptide elongation by incorporating the cyclic anhydride of Fmoc-*N*-methylglutamic acid onto the Sieber resin, and to carry out the macrolactamization step at the β-MeO-Tyr-*N*Me-Ala linkage. The ester bond was formed once the DiMePyroGlu was already coupled and using Alloc-*N*Me-Ala-OH and MSNT in conjunction with *N*-methylimidazole. Desmethoxycallipeltin B and other analogs have also been synthesized and biologically evaluated [111,112]. Syntheses for the linear callipeltins D [113] and E [114,115] have been published too. No bioactivity reported for the described linear callipeltins and the loss of anti-HIV activity for the truncated callipeltin B, strongly point out that the whole “head-to-side-chain” arrangement is crucial to obtain relevant biological activity.

**Figure 7:**
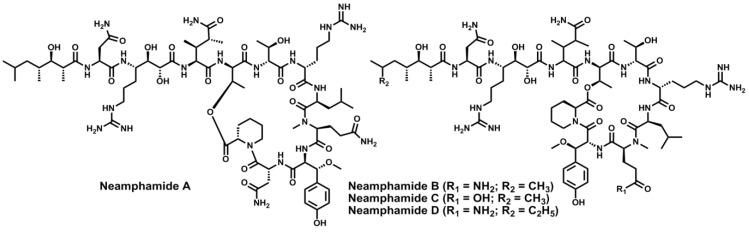
Chemical structures of neamphamides A–D.

**Figure 8:**
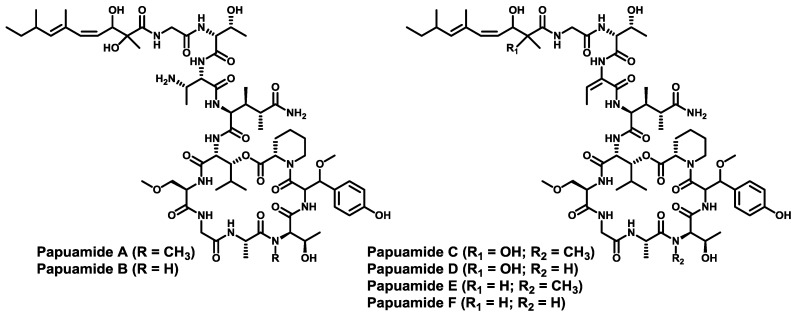
Chemical structures of papuamides A–F. Stereochemical assignment of papuamide E and F has not been published to date. The stereochemistry shown is the one reported after isolation. Differences in the literature are found.

The same new and bizarre three residues were found in other cyclodepsipeptides isolated from the marine sponge *Neamphius huxleyi* in Papua New Guinea. Neamphamide A [116,117], B [118,119], C [119] and D [119] (Figure 7) are depsipeptides with a 25- or a 22-membered macrolactone, a d-*allo*-Thr residue at the branching point and the same terminating polyketide moiety of callipeltin A. Neamphamide A displays a strong cytoprotective effect against HIV-1 infection, while neamphamide B exhibits potent anti-mycobacterial activity against *Mycobacterium smegmatis* and *bovis* BCG, and neamphamides B–D are cytostatic against several human cancer cell lines. An outstanding structural feature and an important difference between neamphamides and callipeltin A is that the former hold the rare l-homoproline in their macrocyclic region. No syntheses have been described so far.

l-Homoproline, β-methoxytyrosine and (3*S*,4*R*)-3,4-dimethyl-l-glutamine are also contained in the papuamides. In 1999, papuamides A–D were isolated in Papua New Guinea from two sponges, *Theonella mirabilis* and *T. swinhoei* [120]. The re-isolation of papuamides C and D along with two new peptides, papuamides E and F, from a marine sponge *Melophlus* sp. was described in 2011 [121]. In addition, papuamides contain other strange amino acids such as d-3-methoxyalanine and (2*R*,3*R*)-3-hydroxyleucine at the branching position, as well as two previously undescribed terminating moieties: 2,3-dihydroxy-2,6,8-trimethyldeca-(4*Z*,6*E*)-dienoic acid and 3-hydroxy-2,6,8-trimethyldeca-(4*Z*,6*E*)-dienoic acid (Figure 8). Papuamides A and B inhibit the HIV-1_RF_ infection of human T-lymphoblastoid cells *in vitro*. Papuamide A also displays cytotoxic activity against a number of human cancer cell lines. The synthesis in solution of papuamide B was published by Ma and co-workers in 2008 [122]. After complete assembly of the macrolactone, it was first coupled to a dipeptide, and the resulting intermediate was then condensate with the last fragment of papuamide B, which comprises the residues d-*allo*-Thr, Gly and the unsaturated α,β-dihydroxy acid blocking the *N*-terminus.

**Figure 9:**
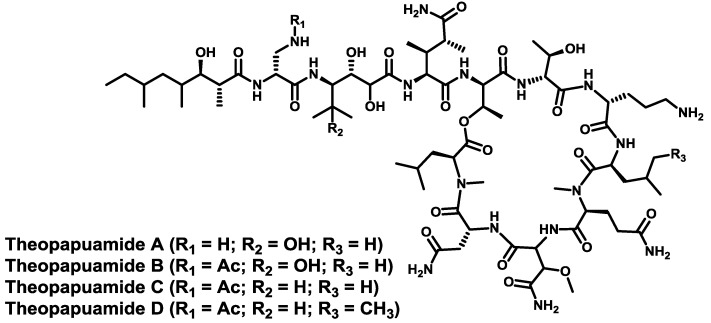
Chemical structures of theopapuamides A–D. No stereochemistry has been described for the residues diMeGln, Amtha and Htoa of theopapuamide A.

From the same *T. swinhoei* sponge in Papua New Guinea, another member of the “head-to-side-chain” cyclodepsipeptides family, namely theopapuamide A [123], was isolated. This is a cyclic depsiundecapeptide bearing a d-*allo*-Thr at the branching position of a 25-membered macrolactone, and two unprecedented amino acid residues in its structure: β-methoxyasparagine and 4-amino-5-methyl-2,3,5-trihydroxyhexanoic acid. The 3-hydroxy-2,4,6-trimethyloctanoic acid acylates the *N*-terminus. Theopapuamides B–D [124] were isolated from the *Siliquariaspongia mirabilis* sponge. They also contain a rare d-3-acetamido-2-aminopropanoic acid and the (3*S*,4*S*)-4-amino-2,3-dihydroxy-5-methylhexanoic acid (Figure 9). Theopapuamides A–C are cytotoxic against human colon carcinoma (HCT-116) cells and show potent anti-fungal activity against wild-type *C. albicans* and also against amphotecirin B-resistant strains. Furthermore, theopapuamide A also exhibits cytotoxicity against the CEM-TART cell line. No syntheses have been described so far.

**Figure 10:**
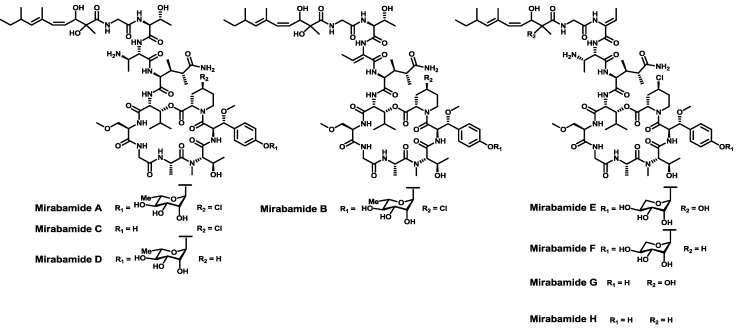
Chemical structures of mirabamides A–H.

Sharing a similar scaffold with papuamides, mirabamides [125,126] were isolated from two different marine sponges *S. mirabilis* and *Stelletta clavosa*. Two unknown building blocks were described after their isolation: the glycosylated amino acid β-methoxytyrosine 4′-*O*-α-l-rhamnopyranoside and the 4-cloro-l-homoproline (Figure 10). Mirabamides A, C and D inhibit HIV-1 in neutralization and fusion assays, thereby showing that their action occurs during early stages of HIV-1 entry. Mirabamides A–C also show anti-microbial activity toward *C. albicans* and *B. subtilis*. No syntheses have been described so far for these structures.

**Figure 11:**
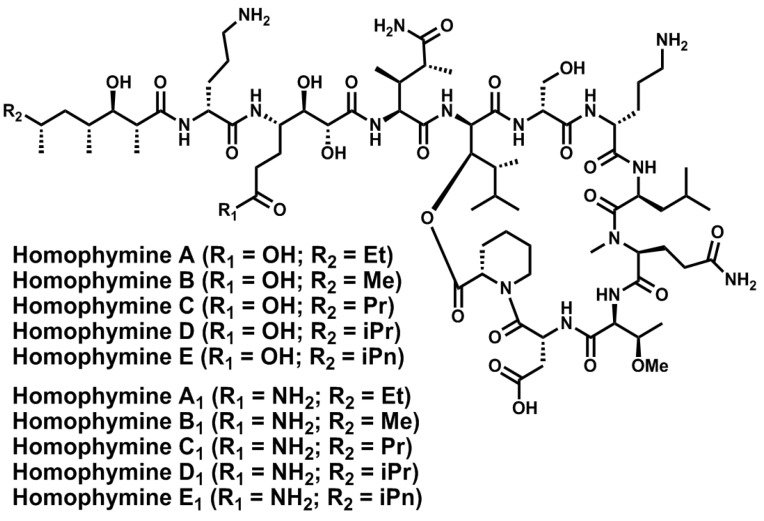
Chemical structures of homophymines A–E and A1–E1.

In 2008 and 2009, Zampella and co-workers described ten new cyclodepsipeptides, named homophymines A–E and A1–E1 [127,128,129], isolated from the marine sponge *Homophymia*, in the order *Lithistida*, collected in New Caledonian. They are all “head-to-side-chain” cyclic depsiundecapeptides that contain: a 25-membered macrolactone; the unusual residues (3*S*,4*R*)-3,4-dimethyl-l-glutamine, l-ThrOMe and l-homoproline; up to five distinct polyketide terminating moieties; and three unprecedented amino acids, (2*R*,3*R*,4*S*)-4-amino-2,3-dihydroxy-1,7-heptandioic acid (ADHA) or (2*R*,3*R*,4*S*)-4,7-diamino-2,3-dihydroxy-7-oxoheptanoic acid (DADHOHA) and the (2*R*,3*R*,4*R*)-2-amino-3-hydroxy-4,5-dimethylhexanoic acid (d-*allo*-AHDMHA) at the branching position (Figure 11). All the members described so far exhibit potent cytotoxic activity. Furthermore, the first isolated member, homophymine A, also displays considerable cytoprotective activity against HIV-1 infection at very low concentrations. No syntheses of these compounds have been described so far.

**Figure 12:**
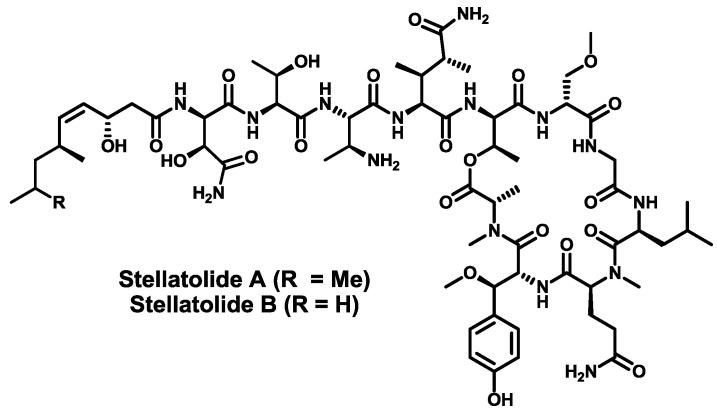
Chemical structures of stellatolide A and B.

In 2010, Pharma Mar isolated two new “head-to-side-chain” depsiundecapeptides, stellatolide A and B (Figure 12), from a marine sponge of the family Ancorinidae, genus *Ecionemia*, species *Ecionemia acervus* Bowebank 1864, collected in Tulear, Madagascar [130]. The only difference between the two peptides is the *N*-terminus acylating moiety. They accommodate in their structures the unusual residues (2*R*,3*R*)-β-methoxytyrosine, (3*S*,4*R*)-3,4-dimethyl-l-glutamine, (2*S*,3*S*)-2,3-diaminobutyric acid, (2*R*,3*S*)-β-hydroxyasparagine and the terminating moieties 3-hydroxy-6,8-dimethylnon-(4*Z*)-enoic acid or 3-hydroxy-6-methylnon-(4*Z*)-enoic acid. Both compounds have proven to display strong antiproliferative activity against three human cancer cell lines (Lung-NSCLC A549, Colon HT-29 and Breast MDA-MB-231).

**Figure 13:**
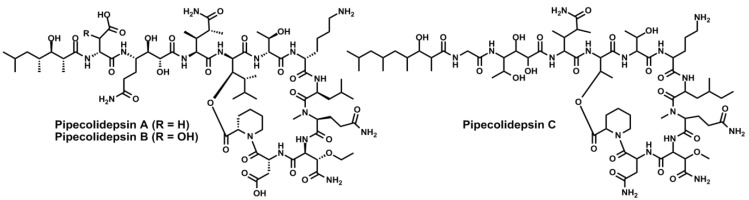
Chemical structures of pipecolidepsins A, B and C.

Finally, from a *Homophymia* marine sponge collected on the coasts of Madagascar, PharmaMar isolated pipecolidepsins A, B and C [131]. These compounds are “head-to-side-chain” cyclic depsiundecapeptides that also bear a 25-membered macrolactone and contain the rare amino acids l-homoproline, l-homoisoleucine, β-EtO-Asn, β-MeO-Asn, (3*S*,4*R*)-3,4-dimethyl-l-glutamine, DADHOHA and the terminating HTMHA acid (Figure 13). Pipecolidepsins A and B have the unique (2*R*,3*R*,4*R*)-2-amino-3-hydroxy-4,5-dimethylhexanoic acid (d-*allo*-AHDMHA) at the branching position, while pipecolidepsin C has a much more simple d-*allo*-Thr residue. The three depsipeptides show relevant cytotoxicity against three human cancer cell lines (Lung-NSCLC A549, Colon HT-29 and Breast MDA-MB-231) with GI_50_ around 10^−7^ M.

## 4. Conclusions

“Head-to-side-chain” cyclodepsipeptides show fascinating chemical structures and remarkable biological profiles, mostly as cytotoxicity. All of them are from marine origin and contain highly complex unprecedented amino acids, as well as, d- and/or *N*Me-amino acids. The distinctive “head-to-side-chain” scaffold via an ester bond has being strongly pointed out as a critical feature responsible for the biological properties displayed by these natural products. Being a synthetic challenge and owing relevant bioactivities have boosted the interest of medicinal chemistry researchers to develop robust and efficient synthetic methodologies to bring these compounds or their analogs to clinical phases.
